# Ethnic and socioeconomic variation in incidence of congenital heart defects

**DOI:** 10.1136/archdischild-2016-311143

**Published:** 2016-12-16

**Authors:** Rachel L Knowles, Deborah Ridout, Sonya Crowe, Catherine Bull, Jo Wray, Jenifer Tregay, Rodney C Franklin, David J Barron, David Cunningham, Roger C Parslow, Katherine L Brown

**Affiliations:** 1 Population Policy and Practice Programme, UCL Great Ormond St Institute of Child Health, University College London, London, UK; 2 Clinical Operational Research Unit, University College London, London, UK; 3 Cardiac Unit, Great Ormond Street Hospital NHS Foundation Trust, London, UK; 4 Paediatric Cardiology Department, Royal Brompton and Harefield NHS Foundation Trust, London, UK; 5 Birmingham Children's Hospital NHS Foundation Trust, Birmingham, UK; 6 National Institute for Cardiovascular Outcomes (NICOR), London, UK; 7 Paediatric Intensive Care Audit Network (PICANet), Leeds, UK

**Keywords:** congenital heart disease, Epidemiology, ethnicity, socio-economic deprivation, record linkage

## Abstract

**Introduction:**

Ethnic differences in the birth prevalence of congenital heart defects (CHDs) have been reported; however, studies of the contemporary UK population are lacking. We investigated ethnic variations in incidence of serious CHDs requiring cardiac intervention before 1 year of age.

**Methods:**

All infants who had a cardiac intervention in England and Wales between 1 January 2005 and 31 December 2010 were identified in the national congenital heart disease surgical audit and matched with paediatric intensive care admission records to create linked individual child records. Agreement in reporting of ethnic group by each audit was evaluated. For infants born 1 January 2006 to 31 December 2009, we calculated incidence rate ratios (IRRs) for CHDs by ethnicity and investigated age at intervention, antenatal diagnosis and area deprivation.

**Results:**

We identified 5350 infants (2940 (55.0%) boys). Overall CHD incidence was significantly higher in Asian and Black ethnic groups compared with the White reference population (incidence rate ratios (IRR) (95% CIs): Asian 1.5 (1.4 to 1.7); Black 1.4 (1.3 to 1.6)); incidence of specific CHDs varied by ethnicity. No significant differences in age at intervention or antenatal diagnosis rates were identified but affected children from non-White ethnic groups were more likely to be living in deprived areas than White children.

**Conclusions:**

Significant ethnic variations exist in the incidence of CHDs, including for specific defects with high infant mortality. It is essential that healthcare provision mitigates ethnic disparity, including through timely identification of CHDs at screening, supporting parental choice and effective interventions. Future research should explore the factors underlying ethnic variation and impact on longer-term outcomes.

**What is already known on this topic?**

- *Safe and Sustainable: Review of Children's Congenital Heart Surgery Services* noted high congenital heart defect (CHD) rates and demand for CHD surgery in areas with large Asian communities.
- Higher prevalence of CHDs in babies of British Asian compared with non-Asian ethnicity has been reported in some regional studies in England.
- Studies using national paediatric audit data to investigate variation in CHD incidence across multiple ethnic groups in the ethnically-diverse contemporary UK population are lacking.

**What this study adds?**

- We report significant ethnic variation in the incidence of serious CHDs in English and Welsh infants, in national data set.
- We found no evidence of differential access to screening or healthcare but infants of non-white ethnicity were more likely to be living in deprived areas.
- Our findings of significant ethnic differences in CHD frequency are key to informing equitable provision of congenital heart surgery and mitigating disparity.

## Introduction

Congenital heart defects (CHDs) are the most common congenital malformation found in newborns, affecting approximately 6–8 per 1000 live births and are the most frequent cause of infant deaths from birth defects.^1^ ^2^ Understanding the distribution of CHDs in the population is key to understanding the burden of these anomalies, including the factors influencing local case mix and severity, in order to anticipate health needs and provide effective and appropriately targeted services for the prevention and management of these conditions. The observed differences in reported birth prevalence between countries^1^ ^3^ may reflect causal factors, case ascertainment or the effectiveness of healthcare prevention.

The birth prevalence of specific defects varies by racial or ethnic group,^4–7^ and British Asian children have been reported to be at higher risk of complex CHDs than non-Asians.^8^ ^9^ Various factors have been proposed as influencing the association between ethnicity and health, including biology, migration, cultural and lifestyle factors, socioeconomic deprivation and inequitable access to health services.^10^ ^11^ Some authors have suggested that socioeconomic disadvantage and reduced access to diagnostic services underlie ethnic differences in CHD prevalence,^4^ ^12^ ^13^ and deprivation has been associated with higher risk of all congenital anomalies, including CHDs, in the UK.^14^

Although understanding the burden and severity of CHDs is essential to improving the quality of care and longer-term outcomes, few studies have explored ethnic variation in CHD incidence in the diverse contemporary UK population. We report here a population-based analysis, using linked routine audit data from the National Congenital Heart Disease Audit (NCHDA) and the Paediatric Intensive Care Audit Network (PICANet^15^), to investigate by ethnic group the incidence and types of CHD found in babies aged <1 year at the time of their first major cardiac surgery or interventional cardiac catheterisation procedure.

## Methods

Children (n=12 390) aged <1 year who received their first major interventional cardiac procedure in England and Wales between 1 January 2005 and 31 December 2010 were identified in the NCHDA^16^ (formerly the National Institute for Cardiovascular Outcomes Research Congenital/Central Cardiac Audit Database). Using the National Health Service (NHS) number, we linked each child's NCHDA record to individual paediatric intensive care admissions recorded by PICANet to construct a single patient-based data set.^17^ We excluded 1780 infants for whom a linked PICANet record could not be identified, 2634 infants whose CHD or cardiac procedure type did not meet our eligibility criteria or who were born outside England and Wales, and 2626 infants whose index intervention was not between 1 January 2006 and 31 December 2009 (to ensure complete ascertainment for estimation of annual incidence rates (IRs)). A sensitivity analysis comparing children with and without a linked PICANet record suggested that failure to match was more likely for infants with fewer admissions, for example, those with mild CHDs (such as isolated patent ductus arteriosus (PDA)) or receiving catheter interventions.

We defined the index procedure as the first major cardiac intervention; this could be a definitive (‘corrective’) or palliative staging procedure, and was either an interventional cardiac catheterisation or surgical procedure. The NCHDA hierarchical classification algorithms defined a single primary diagnosis for each child.^18^ Babies without a diagnosis of structural CHD and premature babies (born before 37 completed weeks gestation) with an isolated PDA were excluded. An area-based deprivation score (Index of Multiple Deprivation 2010) was derived from each child's residential postcode by PICANet and subdivided into quintiles using national cut-offs.^19^

Ethnicity was recorded in the PICANet database using the detailed 16-category NHS ethnicity code, aggregated into six groups (see online supplementary table S1); this classification is used for the Office for National Statistics (ONS) population estimates by ethnic group. The NCHDA database used a bespoke ethnic classification: Caucasian, Asian, black, Oriental and other (including mixed ethnicity). If ethnicity differed between records for a child, the most frequent ethnic group was assigned; no child was assigned with equal frequency to more than one aggregated (six-category) ethnic group. To determine whether missing data in PICANet could be informed by the NCHDA, we evaluated concordance between the ethnic groups of children with a record of ethnicity in both audits (n=3957).

10.1136/archdischild-2016-311143.supp1 supplementary tables

Descriptive statistics are presented as numbers and percentages; 95% CIs were estimated using the binomial exact method. IRs and incidence rate ratios (IRRs) were estimated by sex and ethnic group for all CHD and each CHD subgroup. To calculate IR, we obtained mid-year population estimates by ethnic group for children in England and Wales from the ONS.^20^

## Results

Our analyses are based on 5350 children whose index intervention took place between 1 January 2006 and 31 December 2009 and for whom we had data about paediatric intensive care unit (PICU) admissions, operations and interventional catheterisations performed from birth throughout the first year of life.

### Ethnic classification

No PICANet record of ethnicity was available for 1223 (22.9%) infants, although the NCHDA recorded ethnic group for 1005 of these, of whom the majority were Caucasian (n=717; see online supplementary table S1). The PICANet categories White, Black, Asian, Chinese, other and mixed were paired with the NCHDA categories of Caucasian, black, Asian, Oriental and other-mixed, respectively (see online supplementary table S2). Cohen's κ statistic for agreement was 0.81 (95% CI 0.79 to 0.83) overall. The sensitivity and positive predictive value (PPV) of each NCHDA ethnic category with respect to the corresponding gold standard PICANet category^21^ were evaluated (see online supplementary table S3). The Caucasian, black and Asian categories in the NCHDA demonstrated good concordance (>75% sensitivity and PPV) with the PICANet categories of White, Black and Asian, respectively, and low risk of misclassification, whereas the remaining paired categories were poorly concordant.

For further analyses, children whose PICANet ethnic group was Chinese, mixed or other were aggregated in an ‘All Other’ ethnic group; children whose ethnicity was missing in PICANet but recorded in the NCHDA as Caucasian, black or Asian were assigned respectively to the White, Black or Asian ethnic groups, and children coded as Oriental or ‘other-mixed’ in the NCHDA were assigned to ‘All Other’ ethnicity. We thus established a data set for further analyses in which 95.9% (n=5132) children had ethnicity data; children were grouped into White (n=3968), Asian (n=604), Black (n=240), All Other (n=320) and ethnicity not known (n=218).

### Characteristics of infants undergoing cardiac intervention

We identified 5350 infants with serious CHDs, of whom 2940 (55%) were boys. Boys comprised over half of affected infants within each ethnic group except for the Black ethnic group (47% boys). We assigned each child to one of 21 specific CHD subtypes according to the primary diagnosis. The proportion of children who had a non-cardiac congenital anomaly associated with CHD, including syndromes, was 21.0% (n=1125) and 10.0% (n=534) of infants were born preterm (before 37 completed weeks gestation). Overall 29.0% children (n=1549) were diagnosed antenatally with CHD and the median age at index procedure was 61 (IQR 10–151) days. No significant ethnic differences in these characteristics were identified (table 1).

**Table 1:** Characteristics of children in the data set by ethnic group (n=5350)

	White			Asian			Black			All Other			Ethnicity not stated		
	N=3968			N=604			N=240			N=320			N=218		
Ethnic group	n	% of 3968	95% CI	n	% of 604	95% CI	n	% of 240	95% CI	n	% of 320	95% CI	n	% of 218	95% CI
Sex *(not recorded n=2)*															
Male (n=2940)	2207	55.6	54.0–57.1	332	55.0	50.9–59.0	113	47.1	**40.6–53.6**	166	51.9	46.2–57.5	122	56.0	49.1–62.7
Gestation *(not recorded n=1681)*															
Preterm (n=534)	391	9.9	8.9–10.8	64	10.6	8.3–13.3	25	10.4	6.9–15.0	35	10.9	7.7–14.9	19	8.7	5.3–13.3
Non-cardiac congenital anomalies *(not recorded n=0)*															
Non-cardiac congenital anomalies (n=1125)	823	20.7	19.5 –22.0	132	21.9	18.6 –25.4	60	25.0	19.7 –31.0	83	25.9	21.2 –31.1	27	12.4	8.3 –17.5
Antenatal diagnosis *(not recorded n=278)*															
Antenatally diagnosed (n=1549)	1113	28.0	26.7 –29.5	178	29.5	25.9 –33.3	85	35.4	29.4 –41.8	89	27.8	23.0 –33.1	84	38.5	32.0 –45.3
Age at index procedure *(not recorded n=0)*															
Median age (IQR)	61.5 (10–149) days			56.0 (11–165) days			92.5 (13–168) days			65.5 (12–147.5) days			37.5 (8–132) days		

95% CI indicates 95% CIs using binomial exact method. Bold values indicate 95% CI do not overlap with White reference group.

### Socioeconomic deprivation

**Figure 1:**
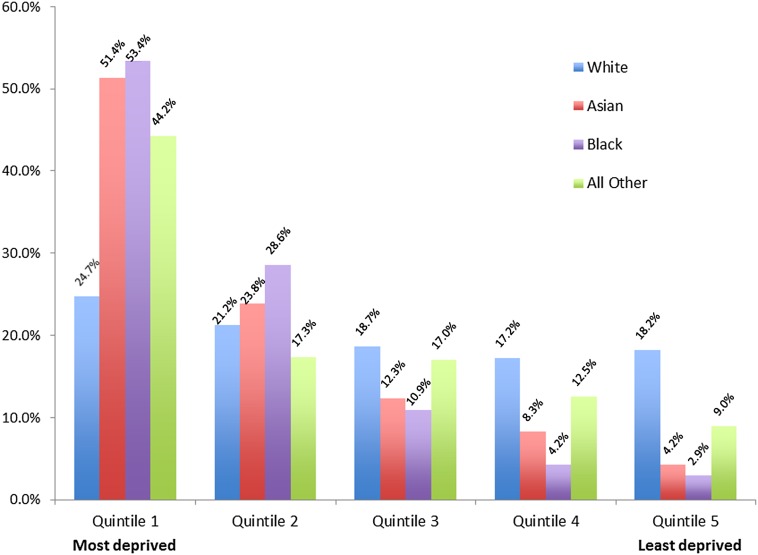
Distribution of cases by ethnic group and deprivation quintile (n=4860). Notes: *Excludes 218 children whose ethnic group was not recorded (from Quintile [Q] 1=52, Q2=46, Q3=37, Q4=30, Q5=37, Quintile not known=16) and 272 additional children whose area deprivation score was not recorded (White=250, Asian/Black/Other=22).

A higher percentage of children with CHD in non-White ethnic groups were more likely to live in the most deprived postcode areas of England and Wales. While 23.2% of White children in the study sample lived in quintile 1 (most deprived), 51.4% of Asian children, 53.4% of Black children and 44.2% children from All Other ethnic groups were resident in areas within the most deprived quintile (figure 1). There were also variations within aggregated ethnic groups (not shown); for example, a higher proportion of children of Asian Pakistani and Asian Bangladeshi ethnicity were living in the most deprived areas compared with those of Asian Indian ethnicity (64.3%, 56.0% and 35.8%, respectively).

### Incidence of CHDs

**Figure 2:**
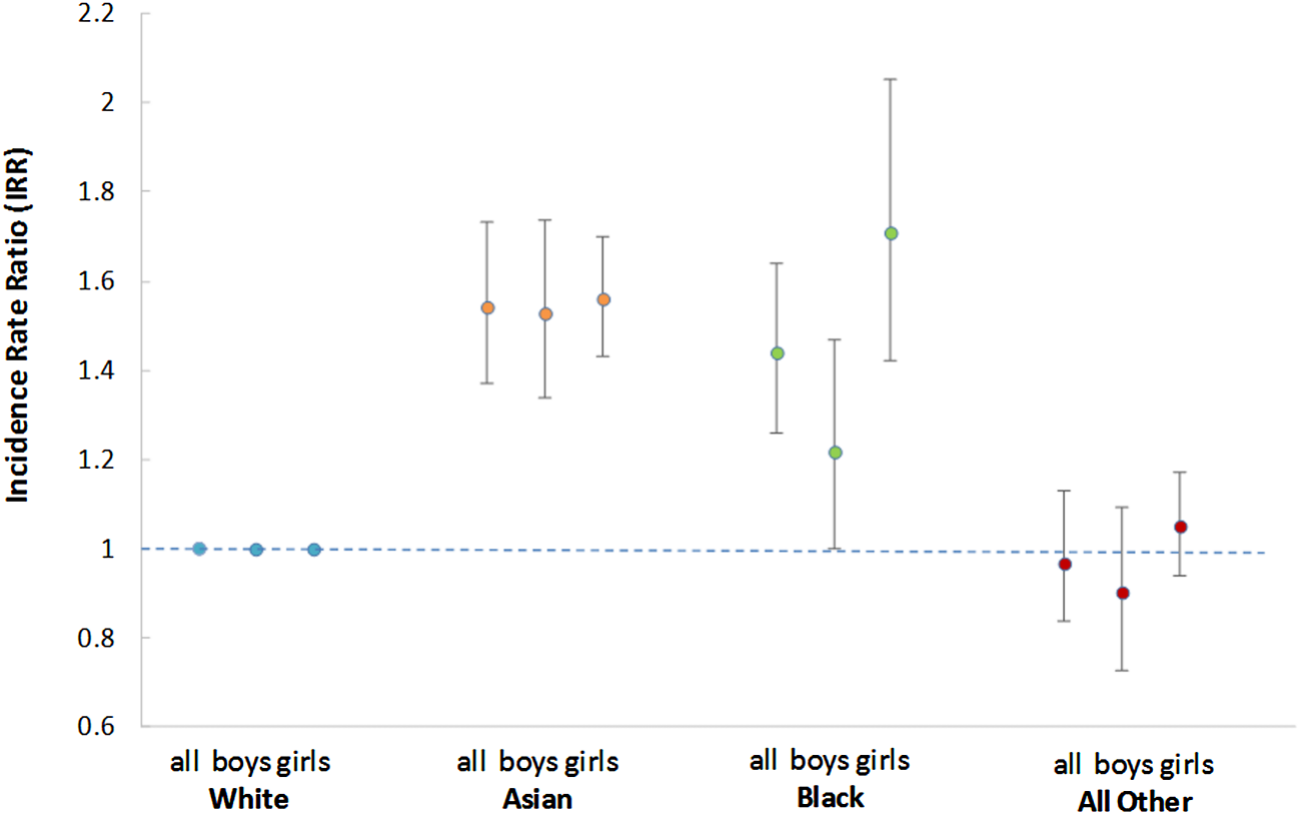
Incidence rate ratios of infants undergoing a cardiac intervention in England and Wales during 2006–2009 by ethnic group (n=5132). Notes: *Excluding 218 children with no ethnicity record; binomial exact method used to estimate confidence intervals for incidence rate ratios (IRR); reference category: White; cardiac intervention includes surgical and interventional catheter procedures.

The incidence of children with structural CHDs who underwent a procedure within the first year of life in the UK during 2006–2009 was 2.0 (95% CIs 1.9 to 2.0) per 1000 children aged <1 year (n=5350 infants) and did not vary significantly by year (see online supplementary table S4). The incidence was significantly higher in children of Asian and Black ethnicity (IRR 1.5 (95% CI 1.4 to 1.7) and 1.4 (95% CI 1.3 to 1.6), respectively) in comparison with the reference White ethnic group (table 2; figure 2). There were no significant variations by sex within ethnic groups (figure 2).

**Table 2:** Incidence rates and rate ratios by ethnic group (for all children undergoing index cardiac procedure, including interventional catheterisation, 2006–2009)

	CHD cases undergoing procedure	Mid-year population aged <1 year*	Annual incidence†	Incidence rate ratio (95% CI)
Ethnic group‡ (n=5132)				
White§	3968	2 230 400	1.8 (1.7–1.8)	Reference
Asian¶	604	220 100	2.7 (2.5–3.0)	1.5 (1.4–1.7)
Black**	240	93 700	2.6 (2.2–2.9)	1.4 (1.3–1.6)
All Other††	320	185 500	1.7 (1.5–1.9)	1.0 (0.9–1.1)

*Mid-year population denominator by ethnic group for infants aged birth to 1 year in England and Wales.^20^

†Values are per 1000 infants with 95% CIs.

‡Rates not reported for 218 children for whom ethnicity was not stated in either source data set.

§Includes children with ethnicity missing in PICANET but classified as Caucasian in the NCHDA.

¶Includes children with ethnicity missing in PICANET but classified as Asian in the NCHDA.

**Includes children with ethnicity missing in PICANET but classified as black in the NCHDA.

††Includes Chinese, mixed and other ethnicity (in PICANET) and children with ethnicity missing in PICANET but classified as Oriental or other in the NCHDA.

CHD, congenital heart defect; NCHDA, National Congenital Heart Disease Audit; PICANET, Paediatric Intensive Care Audit Network.

The incidence of specific CHD subtypes varied by ethnic group (see online supplementary table S5). Compared with the White reference group, children of Asian ethnicity had significantly higher IRs of functionally single ventricle, transposition of the great arteries (TGAs), pulmonary atresia (PA), tetralogy of Fallot, total anomalous pulmonary venous connection, ventricular and atrial septal defects (ASDs) and PDA; children of black ethnicity had higher rates of hypoplastic left heart (HLH), UVH, complete atrioventricular septal defects (AVSDs) and ventricular septal defects (VSD). ASDs were more common in the All Other ethnic group (table 3).

**Table 3:** Incidence rate ratio (IRR) for each congenital heart defect (CHD) type by ethnic group for infants undergoing index interventional catheterisation or surgical procedure, 2006–2009 (n=5132*)

Ethnic group	White		Asian				Black				All Other			
Sample size	N=3968		N=604				N=240				N=320			
Population	N=2 230 400		N=220 100				N=93 700				N=185 500			
	n	IR	n	IR	IRR	95% CI	n	IR	IRR	95% CI	n	IR	IRR	95% CI
Hypoplastic left heart syndrome	231	1.0	30	1.4	1.3	0.9–1.9	22	2.3	**2.3**	**1.4–3.5**	17	0.9	0.9	0.5–1.4
Functionally univentricular heart	193	0.9	41	1.9	**2.2**	**1.5–3.0**	16	1.7	**2.0**	**1.1–3.3**	11	0.6	0.7	0.3–1.3
Common arterial trunk	72	0.3	8	0.4	1.1	0.5–2.3	<5	_	_	_	9	0.5	1.5	0.7–3.0
TGA with VSD/DORV-TGA type	354	1.6	44	2.0	1.3	0.9–1.7	11	1.2	0.7	0.4–1.3	30	1.6	1.0	0.7–1.5
Interrupted aortic arch	51	0.2	5	0.2	1.0	0.3–2.5	<5	_	_	_	<5	_	_	_
TGA with intact ventricular septum	128	0.6	27	1.2	**2.1**	**1.4**–**3.3**	<5	_	_	_	13	0.7	1.2	0.6–2.2
Pulmonary atresia+intact ventricular septum	104	0.5	22	1.0	**2.1**	**1.3**–**3.4**	<5	_	_	_	6	0.3	0.7	0.3–1.6
Pulmonary atresia+VSD (including Fallot type)	129	0.6	23	1.0	**1.8**	**1.1**–**2.8**	9	1.0	1.7	0.7–3.3	17	0.9	1.6	0.9–2.6
Miscellaneous primary cardiac diagnoses (rare)	222	1.0	33	1.5	1.5	1.0–2.2	11	1.2	1.2	0.6–2.2	13	0.7	0.7	0.4–1.2
Complete AVSD	360	1.7	36	1.6	1.0	0.7–1.4	37	3.9	**2.4**	**1.7**–**3.4**	26	1.4	0.9	0.6–1.3
Fallot's tetralogy/ DORV-Fallot type	416	1.9	80	3.6	**1.9**	**1.5**–**2.5**	16	1.7	0.9	0.5–1.5	29	1.6	0.8	0.6–1.2
Aortic valve stenosis (isolated)	106	0.5	8	0.4	0.8	0.3–1.6	<5	_	_	_	<5	_	_	_
Tricuspid valve abnormality (including Ebstein's anomaly)	35	0.2	5	0.2	1.4	0.4–3.7	<5	_	_	_	<5	_	_	_
Mitral valve abnormality (including supravalvar, subvalvar)	38	0.2	7	0.3	1.9	0.7–4.2	<5	_	_	_	7	0.4	2.2	0.8–5.0
Totally anomalous pulmonary venous connection	90	0.4	20	0.9	**2.3**	**1.3**–**3.7**	5	0.5	1.3	0.4–3.2	7	0.4	0.9	0.4–2.0
Aortic arch obstruction±VSD/ASD	467	2.1	54	2.5	1.2	0.9–1.6	19	2.0	1.0	0.6–1.5	23	1.2	0.6	0.4–0.9
Pulmonary stenosis	143	0.6	12	0.5	0.9	0.4–1.5	7	0.7	1.2	0.5–2.5	10	0.5	0.8	0.4–1.6
VSD	661	3.0	111	5.0	**1.7**	**1.4**–**2.1**	55	5.9	**2.0**	**1.5**–**2.6**	66	3.6	1.2	0.9–1.5
ASD	40	0.2	12	0.5	**3.0**	**1.5**–**5.9**	<5	_	_	__	12	0.6	**3.6**	**1.7**–**7.0**
PDA	74	0.3	19	0.9	**2.6**	**1.5**–**4.4**	6	0.6	1.9	0.7–4.4	7	0.4	1.1	0.4–2.5
Miscellaneous congenital terms	41	0.2	<5	_	_	_	<5	_	_	_	<5	_	_	_

*Excluding 218 children with no ethnicity record; IRs per 10 000 infants aged 0–1 years calculated using eligible cases in audit data from 2006–2009; binomial exact method used to estimate CIs for; IRR>1.0 and related CIs shown in bold type. Cell counts <5 are suppressed to reduce disclosure risk. Miscellaneous primary cardiac diagnoses are a group of very rare but severe primary diagnoses, whereas miscellaneous congenital terms comprise miscellaneous structural cardiac defects of varying severity, which are not recognised as distinct primary diagnoses. Isolated subaortic stenosis and aortic regurgitation not shown as ≤10 children per subgroup.

ASD, atrial septal defect; AVSD, atrioventricular septal defect; DORV, double outlet right ventricle; PDA, patent ductus arteriosus; TGA, transposition of the great arteries; VSD, ventricular septal defect.

## Discussion

The incidence of structural CHDs requiring a cardiac interventional procedure during the first year of life is 2.0 per 1000 infants aged up to 1 year in England and Wales. Compared with the White ethnic group, the incidence of all CHDs in Asian and Black infants was around 50% higher and for severe and complex CHD types that have high infant mortality, such as univentricular heart (UVH), HLH, TGA and PA, the incidence in Asian and Black infants was double that for White infants. This IR approximates the birth prevalence for life-threatening structural CHDs as these defects all require an interventional procedure in the first year of life; however, around 5% of children with serious CHDs will die without undergoing any procedure.^22^ The proportion of infants who were diagnosed prenatally and age at first intervention do not vary significantly by ethnicity, thus there was no evidence of differential access to prenatal and postnatal diagnostic services nor clinical intervention. There were also no significant ethnic differences in the proportion of boys, infants born preterm or affected by non-cardiac congenital anomalies; however, children from non-White ethnic groups were more likely to be resident in postcode areas with higher deprivation scores.

Regional UK studies^8^ ^23^ have demonstrated higher relative rates of CHD in British Asian children similar to those evident in our national data set, including increased frequency of complex cyanotic defects and lower frequency of obstructive aortic outflow defects compared with non-Asian babies. Some authors have proposed that consanguinity contributes significantly to increased risk of congenital anomaly,^9^ ^24^ ^25^ particularly within the Pakistani Muslim population; however, a meta-analysis by Bittles estimated the additional anomaly risk due to consanguinity to be around 3%.^26^ Ethnic differences in CHD prevalence have been reported in north America,^4^ ^6^ but these may not be directly applicable to the UK population as the Black and Asian populations in Britain have different migratory and cultural influences to those in the USA.^10^ Nevertheless, international comparisons highlight the complex interplay of biological, environmental and socioeconomic factors, selective migration, cultural and lifestyle influences on the health of different ethnic groups.^10^ ^14^ ^27^ They underline the importance of understanding ethnic diversity and avoiding simple categorisation into white and non-white.^28^

Several authors have highlighted the potential contribution of socioeconomic deprivation to increased CHD risk^14^ and some US authors^4^ ^28^ ^29^ have suggested that differential access to diagnostic health services within a payment-based healthcare system contributes to ethnic disparities in reported CHD prevalence.^12^ ^29^ Conversely, Parslow *et al* have reported that mortality risk for critically ill South Asian children admitted to PICU increased as deprivation decreased.^30^ Nevertheless, British non-White ethnic populations are more likely to be living in more socioeconomically deprived areas;^31^ using our data set, we confirmed that a higher proportion of infants affected by CHDs of Black, Asian and All Other ethnicity were living in the most deprived areas of England and Wales compared with affected white infants.

Importantly prenatal diagnosis may impact on CHD prevalence at birth; in 1999, a prenatal diagnosis of serious CHD was made in 23% of all affected UK pregnancies but only 12% of infants were affected at birth; therefore, affected pregnancies ended in fetal loss or termination in around 50% of cases.^32^ As prenatal detection facilitates parental choice about pregnancy termination, resuscitation and rapid access to surgical intervention after delivery, it will influence birth prevalence, particularly for life-threatening CHDs detected on fetal ultrasound.^33^ In ethnic groups in which the most severe and life-threatening CHDs are more prevalent, pregnancy termination rates might be expected to be higher, or surgical interventions to occur earlier, than in the reference population. Where there are barriers to screening and healthcare services, babies are likely to be older at diagnosis and intervention. Hollowell *et al*^34^ have suggested that UK mothers of Asian Pakistani ethnicity may experience limited access to prenatal screening, and consequently lower rates of pregnancy termination; however, we found no evidence of ethnic variation in antenatal diagnosis rates. Although one study suggested that UK mothers of black ethnicity may be less likely to end a pregnancy affected by spina bifida than those of white ethnicity,^35^ other authors have failed to find evidence of marked ethnic differences in attitudes towards pregnancy termination for congenital anomalies^9^ ^36^ ^37^ and the importance of cultural, religious or ethnic influences therefore remains uncertain.

An important strength of our study was the availability of a linked national data set to support analysis of CHD frequency across a broad range of ethnic groups representative of the contemporary population of England and Wales. However, we could not extend our analysis to Northern Irish and Scottish infants due to the lack of mid-year population estimates by ethnic group for these countries. As submissions to the national audits are mandatory and externally validated, there was good case ascertainment and completion. Nevertheless, some NCHDA records could not be linked to PICANet records, and, although we found no evidence of bias, we cannot exclude the possibility that our findings may have been influenced by missing data. The ethnicity classification currently used by the NCHDA has a significant drawback as it is not comparable with many other data sets, and review of this key variable is now underway to improve monitoring of equity of access to congenital cardiac procedures in future. A further disadvantage of using routine data was that some variables were not collected, including maternal age, and other factors, such as preterm birth or comorbidities, may have been under-reported. As we lacked general population data on ethnic distribution by area deprivation within the age group of interest, we could not assess the relative contribution of deprivation and ethnicity to CHD risk; further research to understand this relationship would be merited. Encouraging improved completion of all variables is therefore crucial to improve data capture from these audits for future analyses, and consideration should be given to exploiting the potential for further linkage to fetal anomaly screening records or congenital anomaly registers, in order to inform understanding of the outcome of conceptions affected by CHDs.

Our study identified important differences in CHD prevalence in England and Wales, and confirmed that infants from Asian and Black ethnic groups are adversely affected compared with the White ethnic group. The reasons for these ethnic variations remain unclear, and, in particular, the relationship between socioeconomic deprivation and ethnicity represents an important focus for future enquiry. Further research into the natural history and outcomes after diagnosis of an affected pregnancy would provide valuable insight into the factors influencing birth prevalence of severe CHDs and postnatal survival to intervention, and would inform the equitable provision of health services to support parental choice during and after pregnancy, as well as the development of interventions to improve the outcomes of all children with CHDs.
