# Supplementary material for: Ethnic and socioeconomic variation in incidence of congenital heart defects
Source: Arch Dis Child. 2016 Dec 16;102(6):496–502. doi: 10.1136/archdischild-2016-311143 (PMC5466927; doi:10.1136/archdischild-2016-311143)
Supplement: supplementary tables [file archdischild-2016-311143supp001.pdf]

List of supplementary tables and figures (Annex 1)

Supplementary Table 1: Detailed ethnic group recorded by PICANet for each individual child

Supplementary Table 2: Comparison of ethnic group recorded for each individual in PICANet and NCHDA for 5350 infants undergoing a cardiac intervention* in England and Wales, 2006-2009

Supplementary Table 3: Agreement between ethnic group categories recorded in PICANet and NCHDA for 5350 infants undergoing a cardiac intervention* in England and Wales, 2006-2009

Supplementary Table 4: Incidence of individual CHD subgroups by ethnic group (infants undergoing a procedure between 2006 and 2009)**

Supplementary Table 5: Annual incidence rate of CHD diagnoses by individual year

Supplementary Table 1: Detailed ethnic group recorded by PICANet for each individual child

Ethnic Group	n
**WHITE (total)**	**3251**
White British	3094
White Irish	11
Any Other White	146
**MIXED (total)**	**146**
Mixed White/Black Caribbean	30
Mixed White/Black African	18
Mixed White/Asian	53
Any Other Mixed	45
**ASIAN (total)**	**444**
Asian/British-Indian	126
Asian/British-Pakistani	185
Asian/British-Bangladeshi	52
Any Other Asian	81
**BLACK (total)**	**160**
Black/British Caribbean	22
Black/British African	107
Any Other Black	31
**CHINESE (total)**	**22**
Chinese	22
**OTHER (total)**	**104**
Any Other Ethnicity	104
**MISSING (total)**	**1223**
Not stated	1223
**TOTAL**	**5350**

Supplementary Table 2: Comparison of ethnic group recorded for each individual in PICANet and NCHDA for 5350 infants undergoing a cardiac intervention* in England and Wales, 2006-2009

**Ethnic Group (PICANet)**	**Ethnic Group (NCHDA)**					
	Caucasian	Black	Asian	Oriental	Other	Missing
White	3068	5	19	*	27	131
Black	6	140	*	*	*	8
Asian	15	*	398	9	5	14
Chinese	*	*	*	16	*	*
Other-Mixed	76	32	48	*	75	16
Ethnicity missing	717	80	160	16	32	218

Notes: *Cardiac intervention includes surgical and interventional catheter procedures. *cells with n<5 are omitted to reduce statistical disclosure risk. Children of mixed ethnicity are recorded as 'Other' in NCHDA, and as a separate 'Mixed' category in PICANet; for this comparison Mixed and Other groups in PICANet were merged (Other-Mixed).

Supplementary Table 3: Agreement between ethnic group categories recorded in PICANet and NCHDA for 5350 infants undergoing a cardiac intervention* in England and Wales, 2006-2009

**Ethnic group (PICANet/NCHDA)**	**Sensitivity:** Percentage children with concordant ethnic group	**PPV:** Percentage children whose NCHDA ethnicity 'correctly' predicts corresponding PICANet ethnicity
White/Caucasian	98.3%	97.1%
Black/Black	92.1%	77.6%
Asian/Asian	92.6%	84.5%
Chinese/Oriental	76.2%	55.2%
Other/Other-Mixed	32.1%	67.2%

Notes: Children of mixed ethnicity are recorded as 'Other' in NCHDA, and as a separate 'Mixed' category in PICANet; for this comparison Mixed and Other groups in PICANet were merged (Other-Mixed category).*Cardiac intervention includes surgical and interventional catheter procedures.

Supplementary Table 4: Annual incidence rate of CHD diagnoses by individual year

**Year of index procedure** *(all infants aged under 1 year)*	**CHD cases undergoing index procedure**	**Mid-year population* aged under 1 year** *(all ethnic groups)*	**Rate per 1000 infants (95% CI)**
**2006**	1250	653400	1.91 (1.81, 2.02)
**2007**	1292	674700	1.91 (1.81, 2.02)
**2008**	1344	702800	1.91 (1.81, 2.02)
**2009**	1464	698800	2.09 (1.99, 2.20)
**2006-2009**	5350	2729700	1.96 (1.91, 2.01)

Note: *Mid-year population estimates from the Office for National Statistics[20]

Supplementary Table 5: Incidence of individual CHD subgroups by ethnic group (infants undergoing a procedure between 2006 and 2009)**

Ethnic Group	White				Asian				Black				All Other			
Sample	N=3968				N=604				N=240				N=22			
Population	N=2230400				N=220100				N=93700				N=19700			
	n	IR*	*95%CI*		n	IR	*95%CI*		n	IR	*95%CI*		n	IR	*95%CI*	
Hypoplastic left heart syndrome	231	1.0	*0.9*	*1.2*	30	1.4	*1.0*	*1.9*	22	2.3	*1.5*	*3.6*	17	0.9	*0.5*	*1.5*
Functionally univentricular heart	193	0.9	*0.7*	*1.0*	41	1.9	*1.3*	*2.5*	16	1.7	*1.0*	*2.8*	11	0.6	*0.3*	*1.1*
Common arterial trunk	72	0.3	*0.3*	*0.4*	8	0.4	*0.2*	*0.7*	<5	-	-	-	9	0.5	*0.2*	*0.9*
TGA with VSD/DORV-TGA type	354	1.6	*1.4*	*1.8*	44	2.0	*1.5*	*2.7*	11	1.2	*0.6*	*2.1*	30	1.6	*1.1*	*2.3*
Interrupted aortic arch	51	0.2	*0.2*	*0.3*	5	0.2	*0.1*	*0.5*	<5	-	-	-	<5	-	-	-
TGA with intact ventricular septum	128	0.6	*0.5*	*0.7*	27	1.2	*0.8*	*1.8*	<5	-	-	-	13	0.7	*0.4*	*1.2*
Pulmonary atresia + intact ventricular septum (IVS)	104	0.5	*0.4*	*0.6*	22	1.0	*0.6*	*1.5*	<5	-	-	-	6	0.3	*0.1*	*0.7*
Pulmonary atresia + VSD (incl. Fallot-type)	129	0.6	*0.4*	*0.7*	23	1.0	*0.7*	*1.6*	9	1.0	*0.4*	*1.8*	17	0.9	*0.5*	*1.5*
Miscellaneous (rare) primary cardiac diagnoses	222	1.0	*0.9*	*1.1*	33	1.5	*1.0*	*2.1*	11	1.2	*0.6*	*2.1*	13	0.7	*0.4*	*1.2*
Complete AVSD	360	1.7	*1.7*	*1.8*	36	1.6	*1.1*	*2.3*	37	3.9	*2.8*	*5.4*	26	1.4	*0.9*	*2.1*
Fallot's tetralogy/ DORV-Fallot type	416	1.9	*1.7*	*2.1*	80	3.6	*2.9*	*4.5*	16	1.7	*1.0*	*2.8*	29	1.6	*1.0*	*2.2*
Aortic valve stenosis (isolated)	106	0.5	*0.4*	*0.6*	8	0.4	*0.2*	*0.7*	<5	-	-	-	<5	-	-	-
Tricuspid valve abnormality (incl. Ebstein's anomaly)	35	0.2	*0.1*	*0.2*	5	0.2	*0.1*	*0.5*	<5	-	-	-	<5	-	-	-
Mitral valve abnormality (incl. supra-, subvalvar)	38	0.2	*0.1*	*0.2*	7	0.3	*0.1*	*0.7*	<5	-	-	-	7	0.4	*0.2*	*0.8*
Totally Anomalous Pulmonary Venous Connection	90	0.4	*0.3*	*0.5*	20	0.9	*0.6*	*1.4*	5	0.5	*0.2*	*1.2*	7	0.4	*0.2*	*0.8*
Aortic arch obstruction ±VSD/ASD	467	2.1	*1.9*	*2.3*	54	2.5	*1.8*	*3.2*	19	2.0	*1.2*	*3.2*	23	1.2	*0.8*	*1.9*
Pulmonary stenosis	143	0.6	*0.5*	*0.8*	12	0.5	*0.3*	*0.9*	7	0.7	*0.3*	*1.5*	10	0.5	*0.3*	*1.0*
VSD	661	3.0	*2.7*	*3.2*	111	5.0	*4.1*	*6.1*	55	5.9	*4.4*	*7.6*	66	3.6	*2.8*	*4.5*
ASD	40	0.2	*0.1*	*0.2*	12	0.5	*0.3*	*0.9*	<5	-	-	-	12	0.6	*0.3*	*1.1*
PDA	74	0.3	*0.3*	*0.4*	19	0.9	*0.5*	*1.3*	6	0.6	*0.2*	*1.4*	7	0.4	*0.2*	*0.8*
Miscellaneous congenital terms	41	0.2	*0.1*	*0.2*	<5	-	-	-	<5	-	-	-	<5	-	-	-

Notes: Incidence rates (IR) per 10,000 infants aged 0-1 years estimated from national audit (2006 to 2009), excluding children with no ethnicity record (n=218); 95% confidence intervals (CI) estimated by binomial exact method; data for subaortic stenosis and aortic regurgitation omitted as disclosive (sample size < 10 children). Cell counts <5 are suppressed to reduce disclosure risk. **Abbreviations:** ASD = atrial septal defect; TGA

= transposition of the great arteries; DORV = Double Outlet Right Ventricle; AVSD = atrioventricular septal defect; PDA = patent ductus arteriosus; VSD = ventricular septal defect.
